# The Fine Structure of Genome Statistics—The Frequency and Size

**DOI:** 10.3390/life15111648

**Published:** 2025-10-22

**Authors:** Piotr H. Pawłowski, Piotr Zielenkiewicz

**Affiliations:** Institute of Biochemistry and Biophysics, Polish Academy of Sciences, 02-106 Warszawa, Poland

**Keywords:** gene number, genome size, density function, intensive and extensive genes, evolution

## Abstract

A determination and mathematical analysis of the statistics of gene numbers in genomes was proposed. It establishes sampling ranges and provides an analytical description of the probability density function, which represents the likelihood of the number of genes in sequenced genomes falling within a specific range of values. The components of the developed statistical multi-Poissonian model revealed the fundamental mechanisms underlying the evolution of life and identified the specific ranges of their dominant influence. The quantitative relations between the statistics of the number of genes and the genome size were shown. A mathematical model of genome size evolution was proposed, identifying subpopulations of intensive and extensive genes associated with protein-coding genes, pseudogenes, and non-coding genes.

## 1. Introduction

The statistical laws of distribution, i.e., Maxwell–Boltzmann [1,2], Planck [3], Bose–Einstein [4], and Fermi–Dirac [5,6], played a significant role in the development of physics. It is timely to ask if the recent achievements of genomic research collected in big databases and statistically modeled could influence modern biology and science as well. A statistical model is associated with an attempt at a mathematical description of the components of the analyzed phenomenon and constitutes an important confirmation of its understanding. The aim of the work is to find the most general characteristics and trends describing comparative genomics.

Seminal studies from the late 20th century, such as the development of the BLAST algorithm [7] and the foundational work by Henikoff and Henikoff (1992) [8], provided essential groundwork for the development of comparative genomics, particularly within the field of bioinformatics. These studies were crucial for understanding the methods and tools that have shaped modern comparative analyses. They improved database searches and the accuracy of protein sequence alignments, which are core techniques in comparative genomics. Later papers in this area include the initial comparative analysis of the mouse [9] and domestic dog genome [10] or the human–chimpanzee genome comparison [11], which are foundational for comparing large genomic datasets, with an understanding of evolutionary relationships and genetic function.

The aim of this study is to analyze the distribution of gene counts and genome sizes within a population of sequenced genomes from organisms representing the three domains of life—Bacteria, Archaea, and Eukaryota—using data collected in the National Center for Biotechnology Information (NCBI [12]) genome database [13].

Basic statistics in functional genomics, such as the distribution of gene counts across known genomes, reveal considerable variation influenced by factors including organismal domain, environmental conditions, genome architecture, and evolutionary history. The vast amount of empirical data available in genomic repositories, analyzed through comparative genomics, provides a detailed understanding of these distributions, highlighting both general trends and notable exceptions [14,15,16].

Typical bacterial genomes contain between about 1000 and 6000 protein-coding genes, with a median of about 3500 [17]. Archaea genomes contain between about 1000 and 5000 genes, with a median near 2500 [18]. Eukaryote genomes are usually above 5000 genes centering around different values, e.g., 10,000 for single-cell organisms [19], 20,000 for animals [20], and 35,000 for plants [19]. In prokaryotes, gene number distribution is unimodal, positively skewed, and centered near 3500 genes with a tail extending toward larger genomes. Eukaryotic gene counts exhibit broader and often multimodal distributions, primarily due to lineage-specific genome duplications and gene losses [20].

In the presented paper, the analysis was based on the determination of a probability density function describing the likelihood of finding any genome failing within a specific range of values of the number of genes, normalized per unit length of this range. The advantage of a proper density function over a histogram is that its values are independent of the sample size.

It was checked that the simple model of genome origination and development as geometric progress is not enough for proper evaluation of the density function and leads to an “ultragenome catastrophe.” In subsequent analyses, accurate results were achieved by modeling the distribution as a linear combination of five Poisson functions, complemented by a single-step function with exponential decay and a constant-level component. This approach allowed us to interpret the observed genome frequency patterns as the outcome of a multi-path evolutionary process, with pathways varying in their rates of change, potential for differentiation, and, in some cases, culminating in gradual elimination. We also show that a simple transformation of gene number statistics can approximately describe genome log (size) statistics. Applied simple regression reveals the characteristic size of genes dominating in the smaller genomes of all domains of life. Extending the regression model to the nonlinear area of the gene number–genome size dependence, we proposed the size-driven mechanism of creation (origination and adding) of a new gene. As presented, the extension contains some speculative elements; it ought to be treated as a conceptual proposal waiting for additional biological validation. The model defines the two distinguished types of genes: extensive ones, which increase genome size when attached, and intensive ones, which do not change the genome size when they are emerging. This provided a compelling explanation for the observed shape of the relationships in the experimental dataset. The theoretical model with fitted parameters could also predict the total fractions of extensive and intensive genes. Finally, based on a successful comparison between estimated ratios and experimental data, we were able to associate extensive genes with protein-coding genes and pseudogenes, and intensive genes with non-coding genes. In general, statistics are often perceived as a tool for lumping all variables into a single analysis. In this study, we aimed to challenge that perception by applying a more nuanced approach.

## 2. Materials and Methods

### 2.1. Genomic Data

The genomic data (Assembly Name, Organism Name, Assembly Stats Total Sequence Length, and Annotation Count Gene Total, Annotation Count Gene Protein-coding, and Annotation Count Gene Pseudogene) for 25,975 reference genomes were taken from the genome database of the National Center for Biotechnology Information (NCBI), (download date: 1 May 2025). Considered genomes, representing Bacteria (eubacteria) (20,931), Archaea (752), and Eukaryota (eukaryotes) (4292), were annotated by NCBI RefSeq or GeneBank submitters and assembled at four genome assembly levels (contig, scaffold, chromosome, and complete). The number of non-coding genes was calculated by subtracting the number of protein-coding genes and pseudogenes from the total number of genes.

### 2.2. Subject of Interest

The subject is the probability density function f_g_, which describes the likelihood of finding a genome with a number of genes falling within a specific range of values, per unit length of that range.

### 2.3. The Data Classification Interval

The range of gene numbers considered spans from 149 to 4,736,081 genes. To classify genomes relative to the increasing number of genes they have, the size of the elementary interval of the class for collecting genomes was examined. For this purpose, the five attempts, with classes of the assumed length of 1, 10, 250, and 500 genes, respectively, were performed. The number of genomes falling in each class was counted to estimate the frequency function. The classes in the pictures were marked by the minimal integer multiples of the interval length, i, they cover, i.e., 0 for the range from 0 to i genes, i for the range from i + 1 to 2i genes, 2i for the range from 2i + 1 to 3i genes, and so on. Next, the maximal values of all attempts divided by the length of the applied class interval (frequency density values) were compared. Then, the attempt with a 500-gene interval was selected as the best representative in the region of the stable values. Thus, the classes in the following pictures were marked by minimal integer multiples of interval length, i = 500, i.e., 0 for 0 to 500 genes, 500 for 501 to 1000 genes, 1000 for 1001 to 1500 genes, and so on.

### 2.4. Probability Density Function f_g_

The probability density function f_g_ was evaluated by dividing the frequency values (for class intervals of 500 genes) by both the length of the class interval and the total number of genomes. Specifically, f_g_[i] = class content[i]/(500 × 25,975).

### 2.5. Mathematical Modeling of Probability Density Function f_g_

#### 2.5.1. Naive Model

A mathematical model for the description of the obtained probability density function was based on the assumption that genome frequency is an exemplification of the process of genome evolution. Initially, we assumed that this process resembles geometric progress (each genome produces q genomes in the higher class) and is therefore described by the function

f_g_[k] = f_g0_q^k^ (1)

where f_g0_ is the initial value, and k is the ordinal number of the class, i.e., the number of subsequent steps in the movement between classes to reach a given class from the zero class in the beginning (for the classes dividing genomes among 500th-gene intervals, k = i/500).

#### 2.5.2. Multi-Poissonian Model

With an unsatisfactory result, the next model was proposed as a linear combination of the shifted Poisson distributions and the uniform distribution of a constant value as background. The number of Poisson distributions considered was gradually increased to reduce the ratio of the mean fitting error to the mean experimental value below 15%. To reach the goal, with the value of the relative error below 20%, the five Poisson functions and constant were effectively supplemented by the step function with the exponential decay, reducing the relative error to 11%. So finally,

f_g_[k] = Σ_(i = 1…5)_ a_i_P_i_[k − k_0i_] + a_s_S[k − k_0s_] + a_b_bg (2)

where P_i_ is the Poisson distribution, S is the step distribution with exponential decay, bg is a constant background distribution, a_i_, a_s_, and a_b_ are coefficients, and k_0i_ and k_0s_ are shifts in the distribution starting along the gene number axis.

The fit of such a model to the evaluated probability density function was the basis of the further discussion. Fitting was performed, predicting the frequency in the integer numbering classes, and dividing the obtained value by 500.

### 2.6. Genome Size

The range of genome sizes considered spans from 112,590 to 40,054,324,612 base pairs (bp). To classify genomes according to the increasing number of base pairs (bp), the size was logarithmized and the length of the elementary interval 0.1 was assumed, compliant with Rice’s rule (number of intervals) = 2 × (number of data points)^1/3^ [21]. The number of genomes falling in each class was divided by 0.1 and next by the total number of genomes to estimate the respective probability density function f_s_ for genome size.

### 2.7. Mathematical Approximation of the Probability Density Function f_s_

A mathematical approximation of the probability density function f_s_ for genome size was obtained due to the transformation:

g ⟶ s = p_1_ g (3a)

f_g_ ⟶ f_s_ = p_2_ f_g_[g] (3b)

where a simple linear regression model of the relationships between gene number, g, and the genome size, s, was assumed (Equation (3a)). The f_g_ and f_s_ are probability density functions for the number of genes and size; p_1_ and p_2_ are parameters.

### 2.8. Extended Mathematical Model of Genome Size Evolution

The evolving genome of size s, and the number of genes g are considered. The relationships between the number of genes and genome size are sought.

**Assumption 1.**  *The small change in the number of genes, the dg, may be divided into two parts, i.e., the part, the dg_e_, containing extensive genes whose presence changes the size of the genome, and the part, the dg_i_, containing intensive genes that do not change the genome size. Thus,*

dg = dg_e_ + dg_i_ (4)

**Assumption 2.**  *An average increase in the genome size, the ds, can be calculated as*

ds = l_e_dg_e_ (5)

*where l_e_ denotes the average change in genome size per extensive gene.*

**Assumption 3.**  *The part of new extensive genes decreases proportionally to l_e_, which may be described as*

dg_e_ = (1 − (l_e_/l_emax_))dg (6)

*where the parameter l_emax_ describes the maximal value l_e_, constrained by molecular and physiological constraints.*

**Assumption 4.**  *Relative to the minimal genome, the average genome size change per extensive gene is proportional to the increase in the actual size of the genome; thus,*

l_e_ = a(s − s_0_) + l_e0_, a > 0 (7)

*where the positive parameter a is a proportionality coefficient, s_0_ denotes the size of the smallest possible genome, and l_e0_ represents the average length of the extensive genes within it.*

Conclusions:

Equations (4) and (6) lead to the conclusion that

dg_i_ = (l_e_/l_emax_)dg (8)

By substituting Equations (6) and (7) into Equation (5), the following expression is obtained:

ds/dg = a((s − s_0_) + b)(1 − c((s − s_0_) + b)) (9)

where

b = l_e0_/a (10)

c = a/l_emax_ (11)

The continuous approximation of Equation (9), after replacing the difference quotient ds/dg with its derivative, s′(i.e., the limit value for dg ⟶ 0), consequently allows for the formulation of the differential equation

s′[g] = a((s[g] − s_0_) + b)(1 − c((s[g] − s_0_) + b)) (12)

the solution of which is the function:

s[g] = Ae^a(g − g^_0_^)^/(1+ Be^a(g − g^_0_^)^) − A/(1 + B) + s_0_ (13)

where g_0_ is the number of genes in the minimal genome, and

A = b/(1 − bc)(14)

B = bc/(1 − bc)(15)

Equation (13) was used as a nonlinear regression model of genome size dependence on the number of genes, which describes the approximate relation between the gene number and the genome size in the full range of considered numbers of genes.

### 2.9. Fitting

All minimizations were performed using Solver (1990–1995), a Microsoft Excel (2007) add-in program [22].

The Microsoft Office Excel Solver add-in is part of a set of commands sometimes called simulation analysis tools. It can be controlled via its user-friendly dialog box or by editing Visual Basic code. The program finds the optimal value for a formula in a single cell—called the target cell—in a worksheet. During this process, it adjusts the values in changing cells you specify—called matched cells—to obtain the user-specified result (minimum, maximum, or defined value) based on the formula in the target cell. The Solver add-in can work with a group of cells related, directly or indirectly, to the formula in the target cell. It uses the Generalized Reduced Gradient (GRG2) nonlinear optimization algorithm [23]. For linear and integer problems, the simplex method with constraints on variables and the branch-and-bound method were implemented. Possible constraints can limit the range of values used in the model and can refer to other cells that affect the formula in the target cell. Part of the source code of the Microsoft Office Excel Solver add-in is proprietary. The guides on how to mimic Solver by the Pulp (3.7.2) Python Package for Linear Programming (3.10.5) may be found on the internet [24,25].

For the purpose of this work, Solver was controlled via the dialog box activated in an Excel worksheet containing the analyzed data. The target cell was defined to represent the square root of the mean square error of fitting of predicted values to the corresponding originals, which was finally minimized. The matched cells represented the parameters of the fitted model used in the calculation of predictions and thus were indirectly related to the target value. Predefined Solver parameters and selected options were as follows: max time 100 s, iterations 100, precision 0.000001, tolerance 5%, convergence 0.0001, tangent estimate, forward derivative, and Newton method.

### 2.10. Estimation of the Total Fractions of Extensive and Intensive Genes, g_e_/g and g_i_/g

An estimation of the fraction of extensive genes g_e_/g in the genomes of a given g was performed on the assumption that all the genomes are arranged according to the increased number of genes and size (second order). Then, for the genome k in the series, the ratio of the total number of attached extensive genes to the total number of all its genes, g_e_/g [g_k_], is performed by the formula

g_e_/g [g_k_] = (Σ_(i = 1…k)_ dg_e_/dg[g_k − 1_](g_k_ − g_k − 1_])/g_k_ (16)

where according to Equations (6) and (7),

dg_e_/dg[g_k − 1_] = (1 − (l_e_[s_k − 1_]/l_emax_)) (17)

l_e_[s_k_] = a(s_k_ − s_0_) + l_e0_ (18)

The value of s_k_ for a given g_k_ was calculated according to Equation (13).

The ratio of the total number of emerging intensive genes to the total number of all genes g_i_/g [g_k_] was performed by the formula

g_i_/g [g_k_] = 1 − g_e_/g [g_k_] (19)

## 3. Results

### 3.1. Determination of the Gene Number Classification Interval

**Figure 1:**
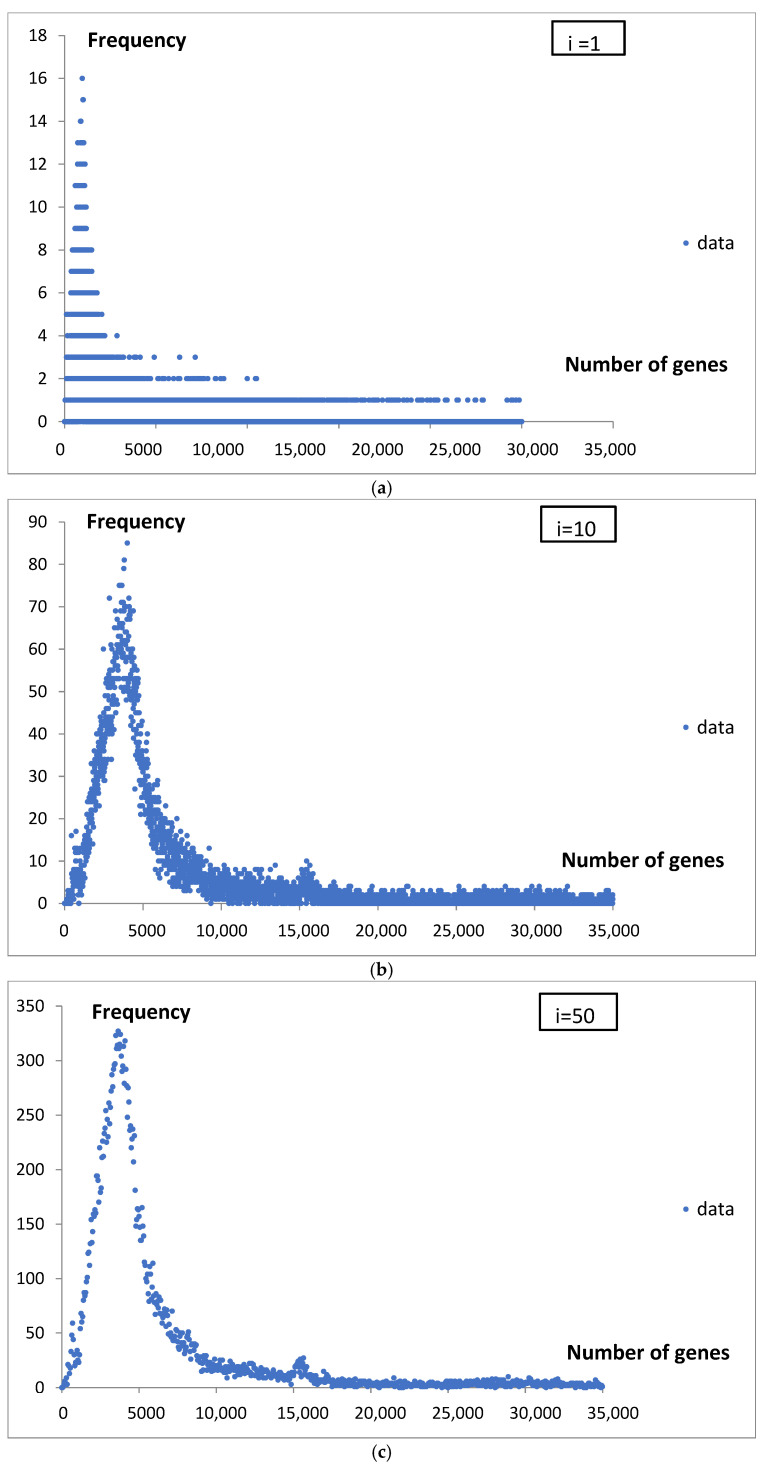

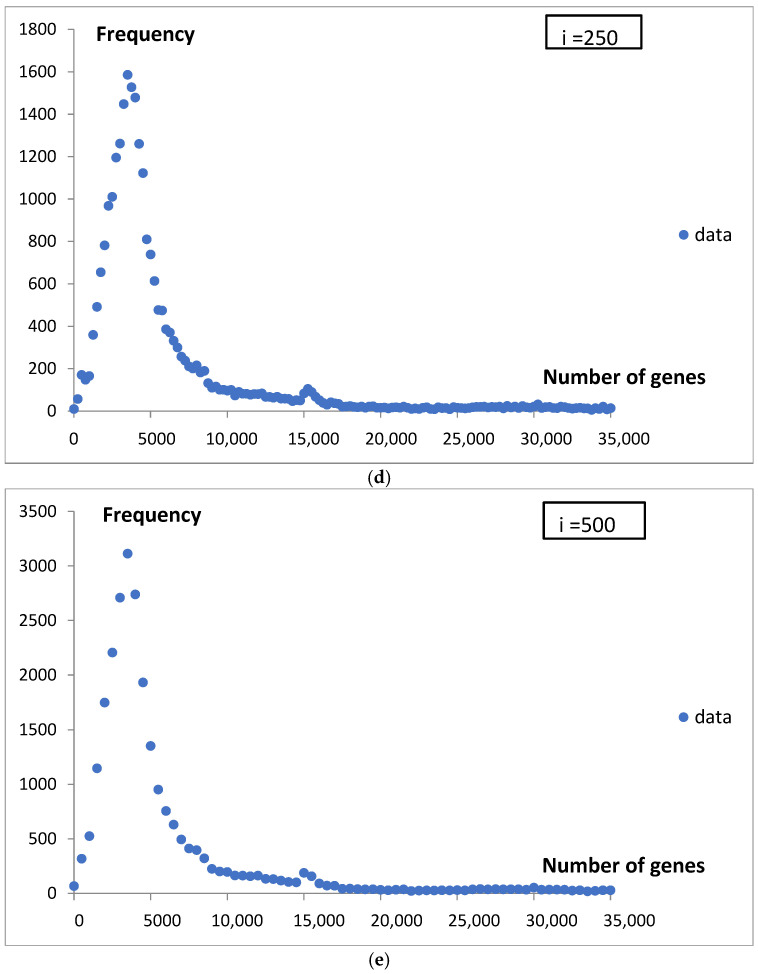
(**a**) The dependence of genome frequency, grouped into classes by gene number, for a classification interval length of i = 1 gene. (**b**) The dependence of genome frequency, grouped into classes by gene number, for a classification interval length of i = 10 genes. (**c**) The dependence of genome frequency, grouped into classes by gene number, for a classification interval length of i = 50 genes. (**d**) The dependence of genome frequency, grouped into classes by gene number, for a classification interval length of i = 250 genes. (**e**) The dependence of genome frequency, grouped into classes by gene number, for a classification interval length of i = 500 genes.

The pattern of genome frequency by gene number was analyzed using different classification interval widths, specifically i = 1, 10, 50, 250, and 500 genes. The data were grouped up to 100,000 genes. The results for the intervals of different lengths are presented in Figure 1a–e. The presentation covers up to 35,000 genes.

**Figure 2:**
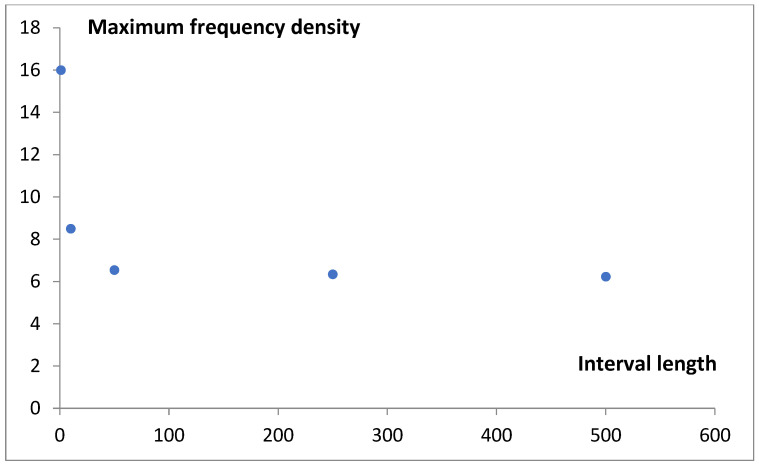
The dependence of the maximum value of the genome frequency density on the length of the classification interval.

The increase in the length of the classification interval stabilizes the estimated frequency density (Figure 2). In order to compute the probability density function for gene number, an interval length of 500 genes was ultimately selected. This corresponds to a resolution of 0.5% (500/100,000 × 100%) relative to the range of grouped gene numbers analyzed.

The data in Figure 1e, divided by the interval length (500) and the total number of genomes (25,975), were used for the estimation of the probability density function for the number of genes, f_g_.

Additional analysis of histogram bin width gives Rice’s rule [21] value hR = 2 × (number of data points)(1/3) = 59 and Freedman–Diaconis rule [26] value hFD = 2 × (Q3 − Q1) × (number of data points)(−1/3) = 225, where Oi means quartile i.

### 3.2. Mathematical Models of Probability Density Function

**Figure 3:**
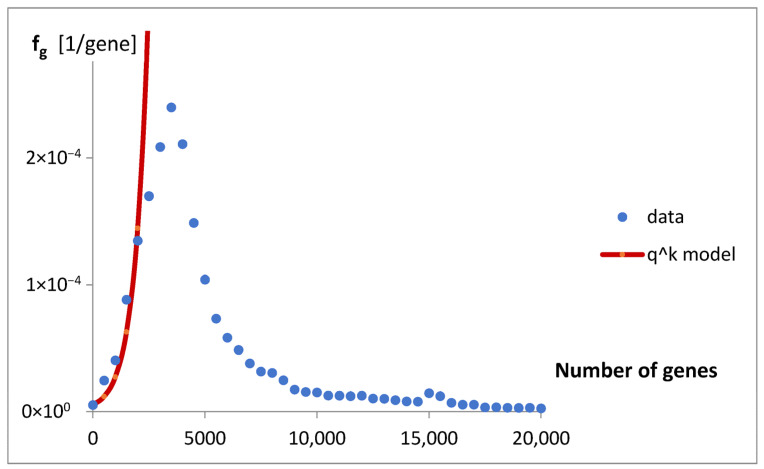
The model q^k^ of geometric progress (f_g_ = f_g0_q^k^) fitted to the estimated probability density function for the number of genes, f_g_. The resulting adjustment parameters are f_g0_ = 5.2 and q = 2.3.

To mathematically describe the obtained result, a simple model of the geometric progression was fitted to f_g_. The landscape of the “catastrophic” prognosis is presented in Figure 3.

**Figure 4:**
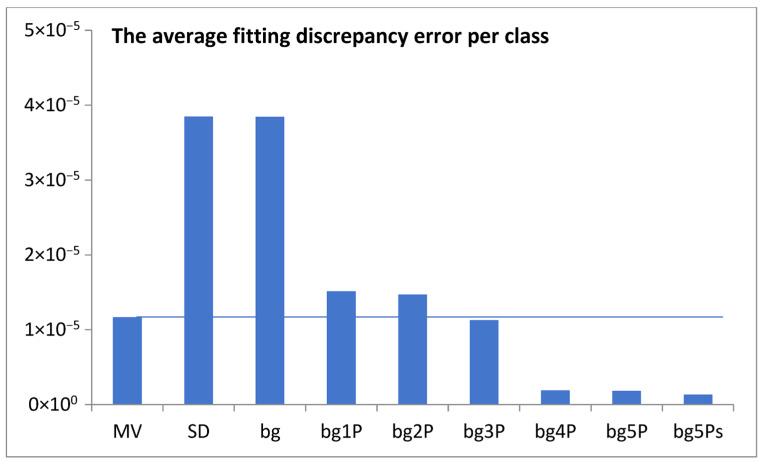
The minimization of the average discrepancy error per experimental point was analyzed with the sequential addition of model components: a background constant (bg), followed by *n* Poisson distributions (bgnP) for up to *n* = 5, and finally a step distribution with exponential decay (bg5Ps). MV is the mean value of experimental data, and SD is the standard deviation of the data.

To achieve a better fit, a multi-component model was examined. Components were added gradually as follows: constant background, plus *n* functions of a shifted Poisson distribution, and the step distribution with exponential decay. The change in the minimization of the average fitting discrepancy error per class (expressed as the root mean square error) with the addition of successive components is shown in Figure 4. The data was taken for the best-fitted model, removing its parts in the opposite direction.

**Figure 5:**
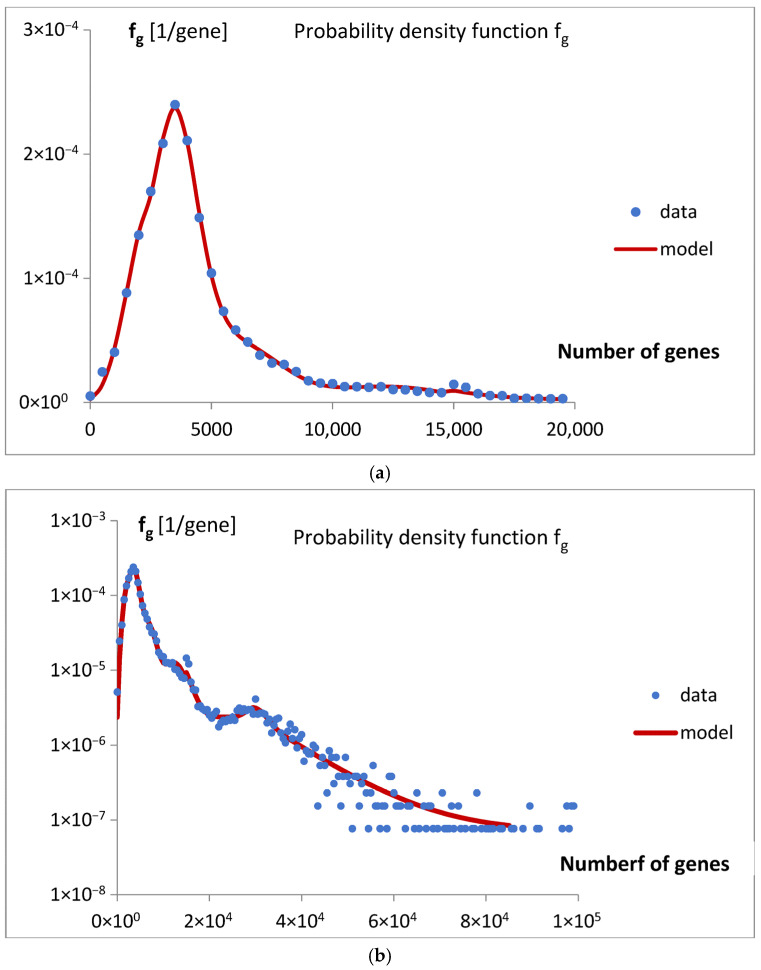
(**a**) The fitting of the mathematical model bg5Ps to the data for probability density function f_g_ is presented for a smaller number of genes. (**b**) The fitting of the mathematical model bg5Ps to the data for probability density function f_g_ is presented across the full range of gene numbers. The f_g_ axis is presented on a logarithmic scale.

The final version of the mathematical model, bg5Ps, was fitted to the probability density function f_g_, and the results are presented in Figure 5a,b.

Parameters of the model are presented in Table 1.

**Figure 6:**
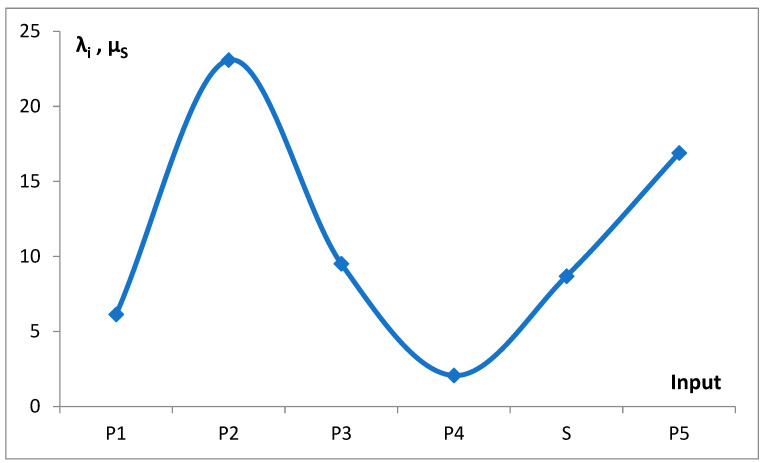
The rate of considered inputs into the distribution of the number of genes. Parameters λ for Poisson inputs P_1_–P_5_ and parameter µ_s_ for the step input were taken from Table 1. An (Excel) smooth line connecting the points was added.

The variation in the rate λ_i_ of Poisson components and the mean value of S input, µ_s_ = (L_s_^2^/2 + 1/γ)/(L_s_ + 1/γ), is presented in Figure 6.

**Figure 7:**
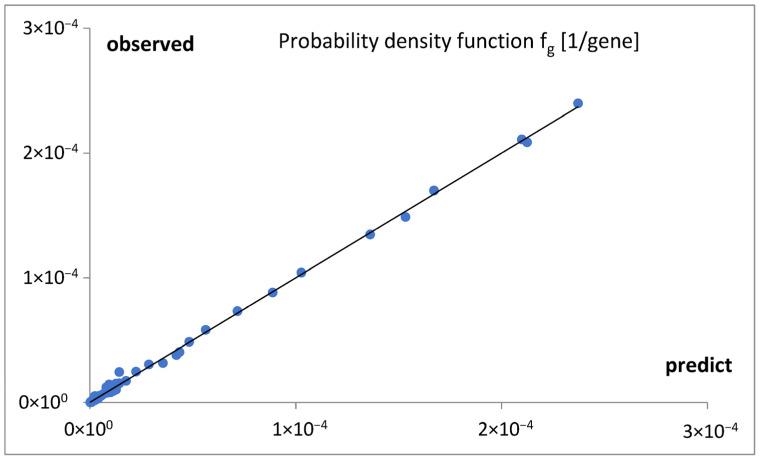
The quality of the fitting (Figure 5a,b) of the mathematical model bg5Ps to the data for probability density function f_g_. A linear trend was shown, y = 0.9994x + 9 × 10^−8^, at R^2^ = 0.9987.

The quality of the fit is documented in Figure 7.

**Figure 8:**
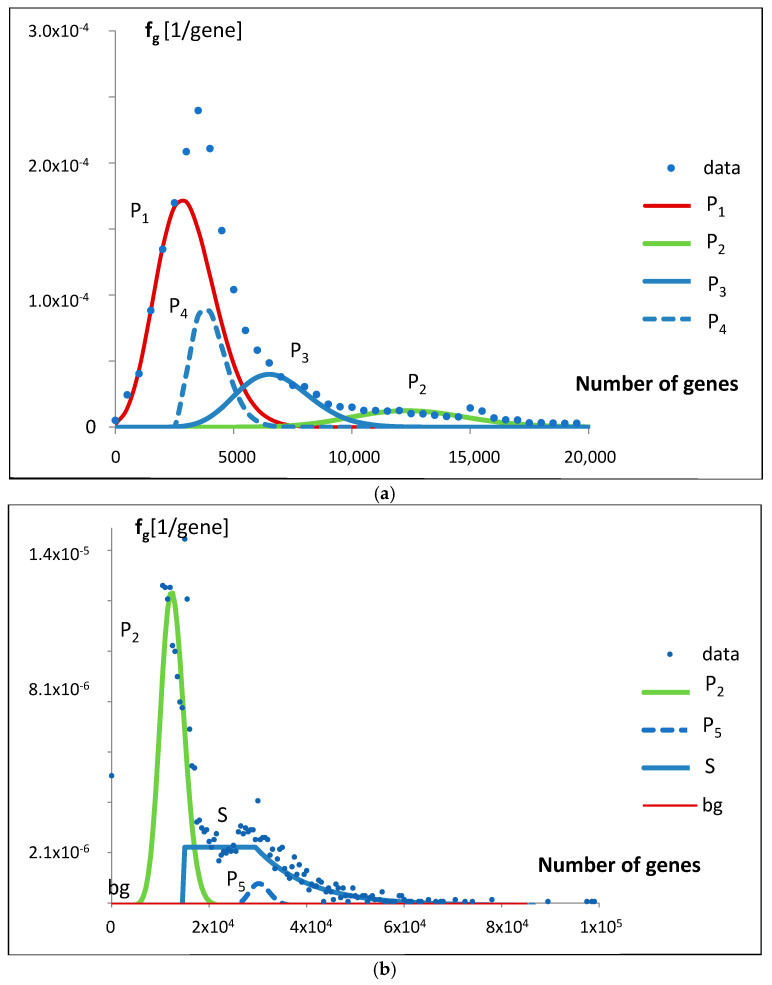
(**a**) The decomposition of the fitting (Figure 5a,b) of the mathematical model bg5Ps to the data for probability density function f_g_, presented for genomes with a smaller number of genes. (**b**) The decomposition of the fitting (Figure 5a,b) of the mathematical model bg5Ps to the data for probability density function f_g_, presented across the full range of the number of genes. The f_g_ axis is presented in a logarithmic scale.

The fitting can be decomposed into the constitutional parts. The result is presented in Figure 8a,b.

**Figure 9:**
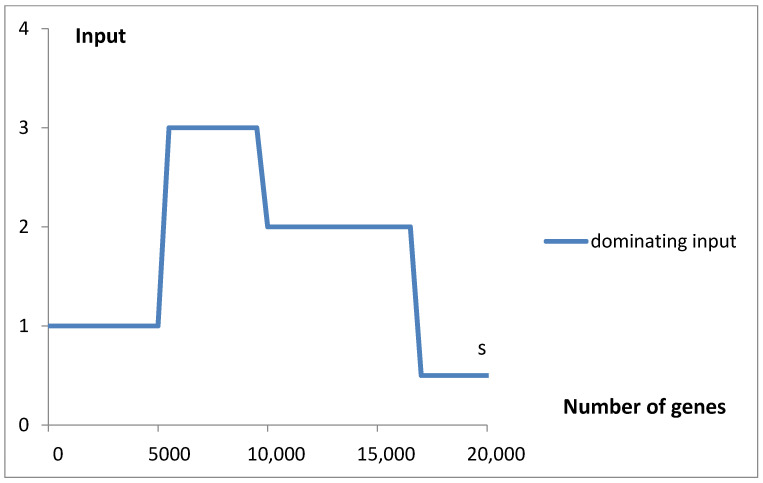
The dominating component of the mathematical model bg5Ps fitted (Figure 5a,b) to the data for the probability density function f_g_ is presented for the full range of the number of genes.

The dominating component input (bg, 1–5 Poisson or step with exponential decay) was signaled in Figure 9.

A hold-out set type validation step was proposed to enhance the confidence of the above model. In this step, the fitting was performed once more, but this time to a smaller dataset, in which the data for the number of genes falling in the range of 3001–4500 were excluded from consideration. This way, 33% (8564/25,975) of initially considered items were omitted. The removed data intentionally represent close vicinity of the predicted maximum possible value of the analyzed density function. In the above test, the obtained new parameters of the model differ from the initial values for the total dataset, on average, by 7% and no more than 27% (as for λ4, Table 1). The predictions of the narrowed model regarding the removed values of the density function (classes 6, 7, and 8) have an error of 11% on average.

### 3.3. The Analysis of Genome Size

#### Introduction

**Figure 10:**
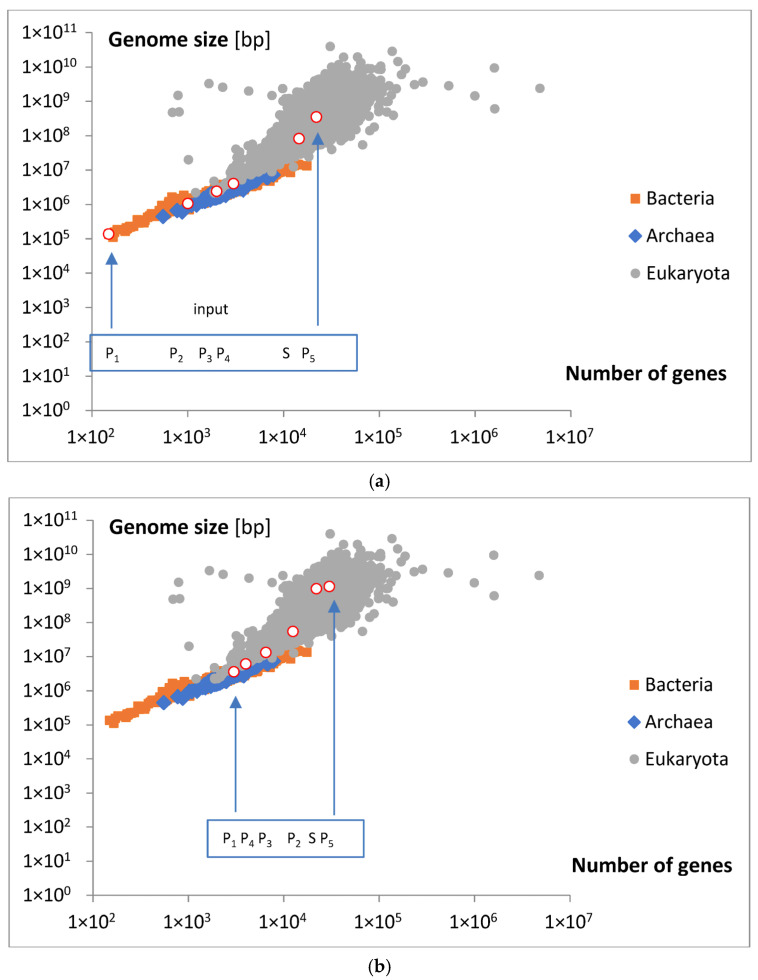

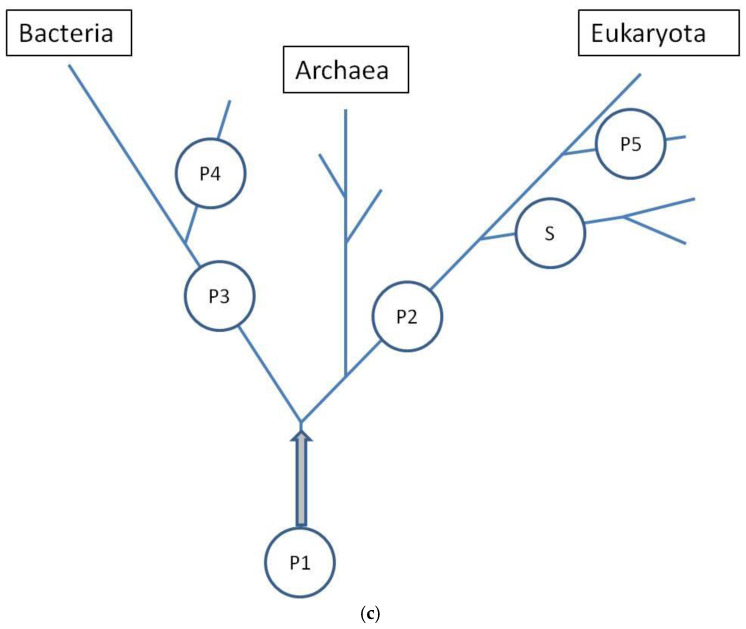
(**a**) The starting points of the subsequent distributions used in the mathematical model bg5Ps, as fitted to the data for the probability density function f_g_ (Figure 5a,b), are presented on the map showing the relationship between the number of genes and genome size. Note that the background data are not classified with respect to the number of genes. (**b**) The points of the maximal input of subsequent distributions used in the mathematical model bg5Ps, as fitted to the data for probability density function f_g_ (Figure 5a,b), are presented on the map showing the relationship between the number of genes and genome size. Note that the background data are not classified with respect to the number of genes. The data for calculating (with Equation (13)) the values of the white-circle points were taken from Figure 8a,b. (**c**) Cartoon showing hypothetical origination of considered inputs on the evolutionary tree.

The presentation of the points of the start of the subsequent distributions and their maximal input are presented on the map of the relation between the number of genes and the size of the genome in Figure 10a,b. Original background data were not classified with respect to the number of genes. The number of genes for starting points (Figure 10a) was attributed using multiplied (×500) data taken from Table 2. The number of genes for maximum points (Figure 10b) was taken from Figure 8a,b. Genome size was calculated according to Equation (13).

**Figure 11:**
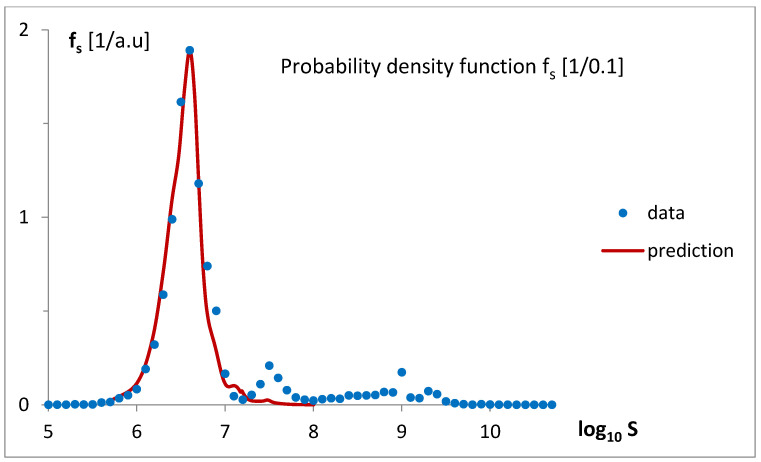
The probability density function for the logarithm of genome size was predicted by transforming the mathematical model bg5Ps, which was fitted to the empirical probability density function f_g_ (Figure 5a,b). In the transformation, proportions between gene number and size and between density functions f_g_ and f_s_ were applied.

The simple linear transformation of the predictions of the mathematical model for f_g_ (Figure 5a,b), using Equation (3a,b) with parameters p_1_ = 1000 and p_2_ = 15.953324, allowed for the estimation of the probability density function for genome sizes, f_s_, presented in Figure 11. A single-point fitting, minimizing the difference between maximal values of f_s_ and prediction, was applied.

**Figure 12:**
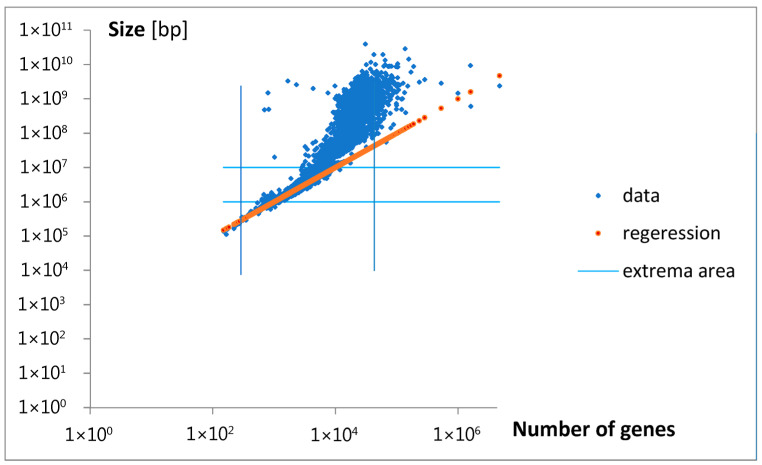
The regression line (red) for the transformation (Equation (3a)) is applied to the estimation of f_s_ in Figure 11. It accurately describes the relation between gene number and genome size, but only within the region around the extrema indicated in Figure 5a and Figure 11, approximately marked by blue lines. Note that presented data are not classified concerning the number of genes.

A visualization of applied regression (Equation (3a)) is presented in Figure 12.

### 3.4. The Genome Size Modelling

#### Mathematical Model

**Figure 13:**
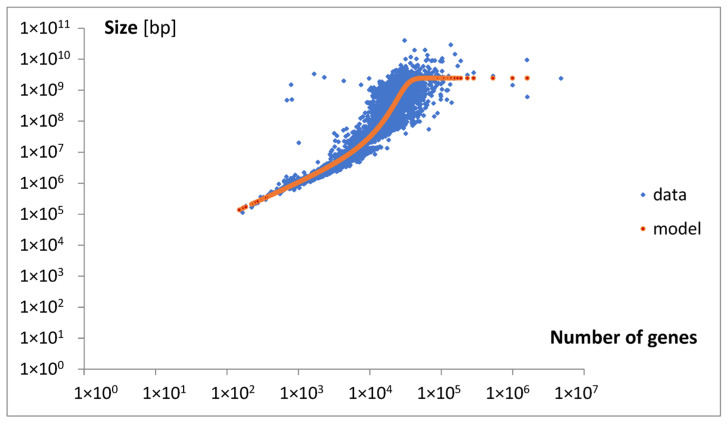
A nonlinear regression model describing the dependence of genome size on the number of genes. It approximates the relationship between the gene number and the genome size across the entire range of gene numbers. The model was fitted to ordinary (not classified) data.

A mathematical model of the relationship between the number of genes and the genome size was proposed (Materials and Methods, Equation (12)), the solution of which (Equations (13)–(15)) was fitted to ordinary (not classified) data. The result of the best fit is presented in Figure 13. The parameters of the best fit are as follows: A, B, a, g_0_, and s_0_. The intermediate parameters are b and c. The final predicted parameters were l_e0_ (Equation (10)) and l_emax_ (Equation (11)). All parameters considered were collected in Table 2.

### 3.5. Intensive and Extensive Gene Analysis

**Figure 14:**
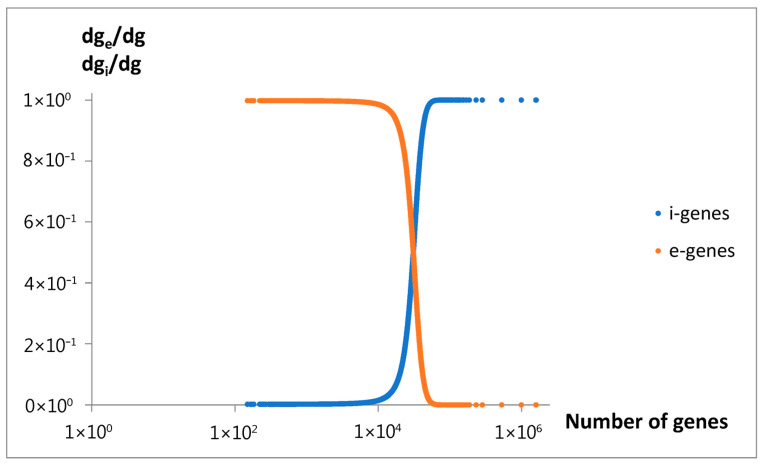
Predicted by the model, the fraction of the number of new extensive genes (e-genes) and new intensive genes (i-genes) changed during evolution vs. the total number of genes. Parameters are shown in Table 2.

The fractions of extensive genes and intensive genes changed in the evolving genome according to Equations (6), (8) and (13), and the parameters from Table 2 are presented in Figure 14.

**Figure 15:**
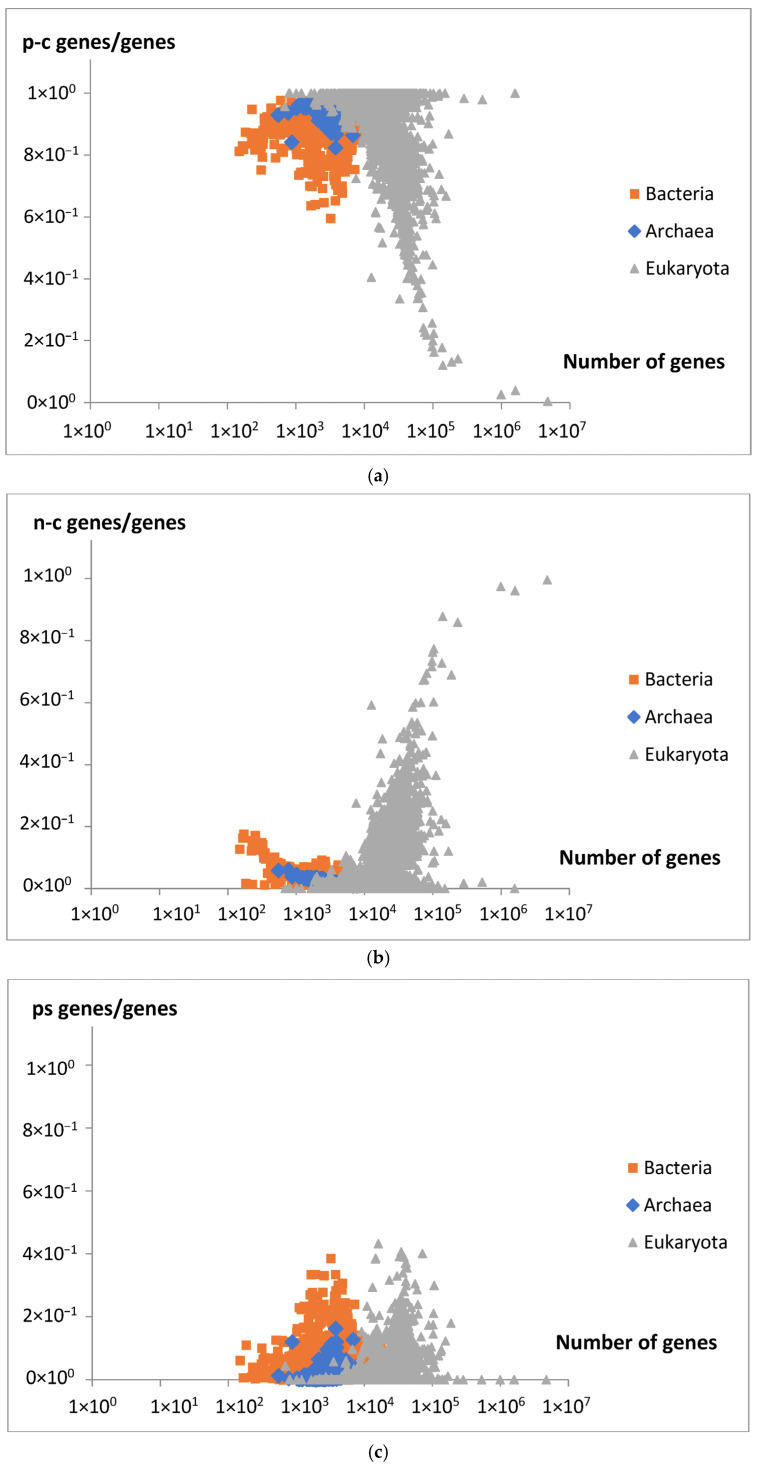
(**a**) The fraction of protein-coding genes (p-c genes/genes) in each genome is plotted against the number of genes in analyzed genomes (25,975). (**b**) The fraction of non-coding genes (n-c genes/genes) in each genome is plotted against the number of genes in analyzed genomes (25,975). (**c**) The fraction of pseudogenes (ps genes/genes) in each genome is plotted against the number of genes in analyzed genomes (25,975).

The dependence of the fraction (the relative number) of protein-coding genes (p-c genes), the non-coding genes (n-c genes), and pseudogenes (ps genes) in a given genome on the number of all genes is presented in Figure 15a–c.

**Figure 16:**
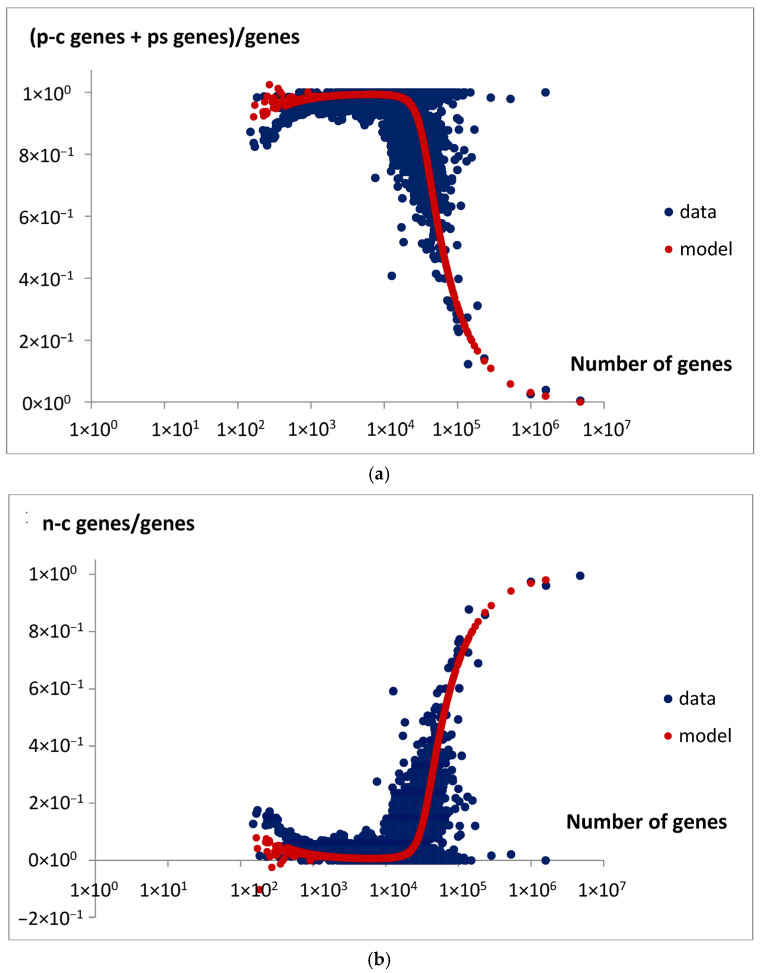
(**a**) The ratio of the sum of pc-genes and ps genes to the number of genes in the genome. The unspecified data (black points) summed up from Figure 15a,c and the predictions (red points indicate fraction of e-genes) of the model (Equation (16)) for parameters listed in Table 2 are shown. (**b**) The ratio of number of nc-genes to the number of genes in the genome. The unspecified data (black points) from Figure 15b and the predictions (red points indicate fraction of i-genes) of the model (Equation (16)) for parameters listed in Table 2 are shown.

The total fractions of extensive and intensive genes, g_e_/g and g_i_/g, were predicted by the model, Equations (16) and (19), according to the parameters from Table 2. Then, they were compared with the data derived, the ratio of the sum of pc-genes and ps genes to all the genes in the genome (Figure 16a), and the ratio of the nc-genes to all the genes in the genome (Figure 16b).

## 4. Discussion

When we analyze genome statistics from large bioinformatics databases, it seems to us that, due to the significant scatter, different sizes of probes, and lack of a mathematical description of the shape, we cannot say much about individual cases, let alone the processes involved in them. Grouping data into classes and determining probability density introduces a standard that can facilitate their discussion and possible comparison with other studies. We illustrated this in Figure 1a–e and Figure 2.

Although the values obtained using Rice’s rule (h_R_ = 59) and the Freedman–Diaconis rule (h_FD_ = 225) support the third and fourth data points in Figure 2, we choose to use a bin width of 500 genes in the histogram analysis. This choice slightly smoothed the data curves, allowing us to focus on the most significant features of the analyzed functions. The subsequent mathematical modeling aimed to deepen our understanding of the nature of the observed statistical distributions.

In the case of genomes analyzed in this paper, to free ourselves from the catastrophic predictions of the geometric model (Figure 3), we sought statistics that would have an inherent ability to limit themselves.

The Poisson distribution models a series of discrete events occurring within a fixed time interval, where λ represents the average number of events (rate) in that interval. The events occur at random, with their exact timing being both independent and memoryless. It decays with the number of events. The exact value of the interval is not important. It may even be infinite.

According to our idea, events can represent genome transitions into subsequent classes containing genomes with an increased number of genes. Thus, the number of genes is treated as a kind of pseudo-time. In our model, on average, the 500 additional genes are significant when considering the latter state. Thus, a chosen group of N genomes, evolving at the rate λ, are currently observed according to a Poisson distribution formed throughout the entire evolutionary period. Moreover, we believe that all genomes initially evolved together, but over time, some groups of them broke off and moved on at different speeds.

This idea was qualitatively described by the model bg5Ps, which was gradually developed and examined in a series of attempts (Figure 4). The final fitting of the model to the data for the probability density function of the gene numbers (Equation (2), Figure 5a,b, Table 1) and predicted oscillating rates of the considered inputs (Figure 6), at the obtained quality (Figure 7), allow us to draw several conclusions.

When decomposing the mathematical model into constitutive components, a landscape of smooth inputs is revealed (Figure 8a), supplemented by a characteristic decaying short jump in the range of larger gene numbers (Figure 8b).

Thus, we can identify five evolving Poissonian groups with the different values of N_i_ and λ_i_, starting at different classes (stages of evolution), and a group appearing as step input with an exponential decay. There is also a uniform background of the order of the standard deviation (Figure 3), which we take for noise of errors. The largest genome content is found in Poissonian group 1, where N_1_ represents a fraction a_1_ = 0.531584 of the total (Table 1), accounting for over 53% of the analyzed population, specifically 13,808 (a1 × 25,975) genomes. Despite this, due to evolutionary dispersion of genome gene numbers, it dominates only in the range of the smallest sets of genes (Figure 9). Genomes with a higher number of genes are predominantly associated with Poissonian group 3. Using Figure 9, with a low probability of error, one may assign *S. cerevisiae* (6477 genes) to the Poissonian group P3 and *H. sapiens* (59,715 genes) to the group S resembling step input.

We believe that Poissonian groups can represent genomes progressing through successive phases of evolution, without preserving less distinct intermediate forms or retaining the forms that quickly disappear. In contrast to this, the step-like group represents genomes, which, when moving from class to class, approximately at the same rate, leave behind replicas of their representatives. However, after a certain number of transitions, the progress of this group exponentially vanishes.

Analyzing the magnitude of the considered inputs, presented in Figure 8a,b, and locating the appearance of the discussed groups on a map illustrating the relationship between gene numbers and genome size, Figure 10a,b, we can approximately conclude that only Poissonian group 1 (P_1_) may contain the smallest bacteria and archaea (Figure 10a). Some genes in this area for eukaryota, with bp > 10^7^, are probably an error. The P_2_ group may contain the smallest and moderate eukaryota. On the other hand, the P_3_ and P_4_ groups may cover moderate and big bacteria (Figure 10b). On the contrary, the S group does not cover any bacteria or archaea but includes moderate and big eukaryota. The last, P_5_ group, contains only moderate eukaryota. Note that the P_2_, S, and P_5_ inputs increased in size more than was predicted by the emerging initial linear regression. To aid in the visualization of these possibilities, hypothetical points of origin for the discussed inputs along the evolutionary tree are illustrated in the schematic shown in Figure 10c.

It is important to acknowledge certain limitations of the above conclusions. Due to overlapping contributions and their theoretically infinite range, it is not possible to establish a flawless correspondence between a class of organisms defined by a given gene number and a single characteristic input. Only genomes with fewer than 500 genes can be strictly related to a single input, P_1_ (neglecting the background error, bg). It is usually a more or less probable relation, so we can only indicate the dominating input among others in a settlement of a certain area of gene numbers. In the opposite direction, search predictability looks better. Only inputs P_1_ cannot be strictly related to one domain of life. Other inputs can be attributed under the assumption of domain inheritance, whereas P_1_ lack any domain-specific attribution.

An example below illustrates the above features in practice. Let us consider a histogram bin with gene numbers in the range 5001–5500. It contains genomes that are 94% bacterial, 1% archaeal, and 5% eukaryotic. On the other hand, the inputs contribute to the total number of genomes in this class, as follows: P_1_-47%, P_3_-23%, and P_4_-30% (Figure 8a). Other inputs may be neglected. One may conclude that P_1_ input delivers 6% genomes for archaea and eukaryota and 41% for bacteria. So, 44% of bacterial genomes in this class are P_1_ type.

As illustrated by the above example, under certain conditions, the input type of a genome belonging to a specific domain and class can be identified, albeit with a relatively high probability of error. The question arises if additional attributes, e.g., kingdoms, phylogeny, lifestyle, and environment, could reduce this uncertainty up to the level of species. This may be an interesting area for separate statistical study, using machine learning methods, especially classifiers.

Finding a mathematical formula for the probability density function, f_g_, regarding gene number, helped in the theoretical determination of the probability density function, f_s_, regarding logarithmized genome size. Fitting the transformed f_g_ (Equation (3a,b)) to the data provides a good estimate of the experimental f_s_ values (Figure 11), but only in the gene number range where the density function exhibits a characteristic dominating bell-shaped peak. The linear regression applied during transformation (Equation (3a)), as illustrated in Figure 12, reveals the limited applicability of this approximation, fortunately confined to the gene number range where genomes occur most frequently, though only among relatively small-sized genomes. The revealed characteristic size per gene (p_1_ = 1000) is in the typical reported range for prokaryotic and small or moderate eukaryotic genomes [27].

In general, the relationship between gene number and genome size is not linear. The first small “acceleration” in the overall genome size may be related to the maximal inputs of the components P_1_, P_3,_ and P_4_ (Figure 10b). The next higher increase falls in the domain of maximal input P_2_. The highest increases may be related to maximal inputs S and P_5_. The appearance of the discussed inputs may also be associated with a strong divergence in genome size at an approximately constant number of genes.

To explain the observed nonlinear behavior, an extended mathematical model of genome size evolution was introduced. The nonlinearity (Equation (13), Figure 13) is interpreted as resulting from a precise fractioning of new genes into two categories: intensive genes (dg_i_), which do not affect genome size, and extensive genes (dg_e_), which contribute to its increase. A key variable of the model used in the constitutive equation (Equation (5)), relating the small change in genome size (ds) with the change in dg_e_, is the average genome size change per extensive gene, l_e_. At the beginning, intensive genes may be related to emerging overlapping genes [28], and extensive genes may be referred to attached genes from duplication [29], or horizontal transfer [30]. According to this model, the fraction of new extensive genes decreases proportionally to the l_e_ (Equation (6)). This assumption describes the self-limiting attaching of large extensive genes, and the consistently increasing emergence of intensive genes (Equation (8)). A basic source determining such effects may be the evolutionary tendency toward minimization of the size of genome and maximization of the number of its genes. Discussed effects could be especially advantaged in the nucleated (eukaryotic) cells, where the mutations producing overlapping genes prevent enormous increase in the size of big genomes. When applying the discussed model, we also have to assume that the average size of attached new material per extensive gene increases with genome size (Equation (7)).

In summary, according to the model, we may expect that with the increase in genome size, the length of new extensive genes increases, but their fraction decreases. This may lead to a slowdown in genome size growth due to the so-called parabola effect. In the extreme case, when the length l_e_ reaches its maximal value l_emax_, genome size expansion may cease entirely. An accompanying increase in the number of intensive genes could further inhibit growth in the total number of genes, ultimately leading to a complete halt. The predicted maximum length, l_emax_, is approximately 498,857.

Approximately analyzing the data in Figure 13 with Equations (6) and (7), we may obtain the following results. For the number of genes around g = 1500 and s = 10^6^, the result is l_e_ = 200, l_e_/l_emax_ = 0.0004, and dg_e_ = 0.9996dg. For g = 20,000 and s = 2.5 × 10^8^, the resulting value is l_e_ = 50,000, yielding l_e_/l_emax_ = 0.1, and dg_e_ = 0.9dg. For g = 50,000 and s = 2.5 × 10^9^, the result is l_e_ = 500,000, l_e_/l_max_ = 0.998, and dg_e_ = 0.002dg.

The discussed model predicts that the minimal length of an extensive gene (l_e0_) is approximately 1009, which is close to the value p_1_. The values g_0_ and s_0_ for the minimal genome used in the model were ultimately set equal to those for the minimal genome in the dataset.

The determined values of the parameters l_emax_ and a (Table 2) are effective for modeling the data across the entire range of gene number variability. In the real case, they may differ for the different groups of genomes and could have been slowly modified during evolution to regulate the rate of genome size increase.

As shown in Figure 6, the dependence of mean values λ_i_ and µ_s_, which characterize the rate of the consecutive inputs to f_g_, on the number of genes exhibits an oscillating pattern. The initial upward trend in the rate of emerging new genes with the increase in the gene number, or the genome size, is nothing special. In light of the discussed findings, the superimposed oscillations, e.g., the slowing of the rate in P_3_ and P_4_, cannot be explained as the result of larger overlapping by an increased number of intensive genes. The mentioned inputs start (Figure 10a) in the region of unnoticeable changes in the intensive gene income (Figure 14). As can be seen, the slowdown in P_5_ by the same mechanism is also doubtful. It is probably because the slight increase in the fraction of new intensive genes cannot seriously modify the ratio of the total number of these genes to all genes. Thus, the reason may be of a more complex evolutionary nature, modifying the fitness of the genome. In this way, it may also produce a rate increase in inputs P_2_ and S. Slow-evolving group P_3_ may contain fungal genomes, and faster-evolving groups P_2_, S, and P_5_ dominate in the gene numbers area of invertebrates, vertebrates, and plants. These kingdoms may have different evolutionary strategies.

The comparison between the model’s genome size predictions and the experimental data supports the model’s validity in accurately describing the overall relationship between gene number and genome size. Furthermore, as predicted by the model, a decrease in a fraction of new extensive genes (Figure 14) is consistent with an observed decrease in the fraction of protein-coding genes (p-c genes) in larger genomes (Figure 15a). It is also consistent with an increasing number of non-coding genes (n-c genes) (Figure 15b), which can be related to the increasing fraction of new intensive genes (Figure 14). Pseudogenes (ps genes), like pc-genes, start to vanish around gene number 3 × 10^4^ (Figure 15c). The results in Figure 15a,b show that the dominant changes concern eukaryota.

The above observations were verified by the predicted total gene pool in the genome. Specifically, the comparison between the predicted fractions of extensive and intensive genes, ge/g (Equation (16)) and gi/g (Equation (19)), and the derived experimental data support this interpretation. These data include the ratio of the sum of protein-coding genes and pseudogenes to the total number of genes in the genome (Figure 16a) and the ratio of non-coding genes to total genes (Figure 16b). This comparison indicates that meaningful relationships between gene types can be established. As suggested, extensive genes that contribute to genome size expansion can be associated with both protein-coding genes and pseudogenes, whereas intensive genes correspond to non-coding genes. Of course, these are not strict rules but rather general observations of dominant trends, which are subject to limitations due to the high dispersion in analyzed data, especially in genomes with large gene counts such as very large eukaryotic genomes. Therefore, they refer to a statistically average situation, and in the specific case, the discrepancy may be particularly large.

Good examples of intensive genes seem to be overlapping genes, especially well-known nested genes. The majority of nested genes are non-coding. For example, in the nematode *C. elegans*, over 92% of nested genes are ncRNAs [31]. Rare examples of coding nested genes are Ins5B and Ins5C in the *E. coli* genome [32], TAR1, NAG1, and CDA12 in *S. cerevisiae* [33], and F8A1 in *H. sapiens* [34].

Extensive genes, on the other hand, can be represented by non-overlapping genes. Approximately 75% of human protein-coding genes were found not to overlap with their neighbors [35]. Although it was shown that pseudogenes may also be related to extensive genes, in fact a significant number of pseudogenes may overlap with protein-coding genes [36]. This overlap may be the result of evolutionary progress in sharing a region of initially distinct gene.

The origins of extensive and intensive genes should be sought in an early stage of life, called the “RNA world” [37,38], which existed before DNA and proteins became dominant. In this world, RNA performed the functions of both modern DNA (informational), proteins (catalytic), and modern RNA (regulational). The early life proliferation of functional RNA molecules required a relatively large operational space for classic non-overlapping sequences, which could not be sufficiently available within a single-stranded RNA. As a result, both the elongating sequences of self-replicating proto-ribozymes and shorter regulatory elements introducing innovations began to overlap, thereby reusing existing sequence space. Host-nested molecule configuration could be evolutionarily preferred, being a precursor of extensive and intensive genes.

Equations (6) and (8) of the model lead to the equation, which may be presented in the form

(l_emax_ − l_e_)dg_i_ = l_e_dg_e_ (20)

describing the balance on a scale with unequal arm lengths (l_emax_ − l_e_) and l_e_, where the “weights” correspond to dg_i_ and dg_e_. Such an analogy may inspire the hypothesis of an equilibrium between the emergence of new intensive and extensive genes, a concept that could be explored in future investigations. In our opinion, future development of the model could also describe, in a more detailed way, the dependence of the length l_e_ on the gene function and a relatively high dispersion of the size of genomes for moderate and high gene numbers.

The authors believe that the aims of this work have been achieved and propose that the analysis of probability density functions, supported by further mathematical modeling, may serve as an effective tool in future bioinformatics research of genomic data, offering valuable insights into the foundations of evolution.

## Figures and Tables

**Table 1:** Parameters of the fitted density function model.

Model Input	Formula	Start Class, k_0i_, k_os_	Parameters L, λ_i_, L_s_, γ	Coefficients a_bg_, a_i_, a_s_
bg	1/L for k ≥ 0	0	200	0.007000
P_1_	λ_i_^k − k0i^e^−λi^/(k − k_0i_)! for k ≥ k_0i_	0	6.133813	0.531584
P_2_		2	23.092150	0.074895
P_3_		4	9.513952	0.153836
P_4_		6	2.071796	0.161564
P_5_		44	16.900607	0.004509
S	1/(L_s_ + 1/γ) for k ≥ 30 and k < 59 e^−γ (k − k0s)^/(L_s_ + 1/γ) for k ≥ 59	30, 59	29, 0.045683	0.058909

**Table 2:** The parameters of the mathematical model of genome size evolution.

Parameter	Definition	Value
A	Fitted	5,000,000.26632
B	Fitted	0.00202752763471099
a	Fitted	0.000202289166662222
g_0_	Fitted	149
s_0_	Fitted	137,475.095259792
b	A/(1 + B)	4,989,883.14035894
c	B/A	4.05505505343353 × 10^−10^
l_e0_	ab	1009.39930220508
l_emax_	a/c	498,856.770121871

## Data Availability

The original data presented in the study are openly available in https://www.ncbi.nlm.nih.gov/datasets/genome/ (accessed on 1 May 2025).
